# Vtc5 Is Localized to the Vacuole Membrane by the Conserved AP-3 Complex to Regulate Polyphosphate Synthesis in Budding Yeast

**DOI:** 10.1128/mBio.00994-21

**Published:** 2021-09-21

**Authors:** Amanda Bentley-DeSousa, Michael Downey

**Affiliations:** a Department of Cellular and Molecular Medicine, University of Ottawa, Ottawa, Ontario, Canada; b Ottawa Institute of Systems Biology, University of Ottawa, Ottawa, Ontario, Canada; Boston Children's Hospital

**Keywords:** AP-3 complex, Apl5, Vtc5, polyP, vacuole, Pep4, ESCRT, VTC complex, *S. cerevisiae*, budding yeast, polyphosphate

## Abstract

Polyphosphates (polyP) are energy-rich polymers of inorganic phosphates assembled into chains ranging from 3 residues to thousands of residues in length. They are thought to exist in all cells on earth and play roles in an eclectic mix of functions ranging from phosphate homeostasis to cell signaling, infection control, and blood clotting. In the budding yeast Saccharomyces cerevisiae, polyP chains are synthesized by the vacuole-bound vacuolar transporter chaperone (VTC) complex, which synthesizes polyP while simultaneously translocating it into the vacuole lumen, where it is stored at high concentrations. VTC’s activity is promoted by an accessory subunit called Vtc5. In this work, we found that the conserved AP-3 complex is required for proper Vtc5 localization to the vacuole membrane. In human cells, previous work has demonstrated that mutation of AP-3 subunits gives rise to Hermansky-Pudlak syndrome, a rare disease with molecular phenotypes that include decreased polyP accumulation in platelet dense granules. In yeast AP-3 mutants, we found that Vtc5 is rerouted to the vacuole lumen by the endosomal sorting complex required for transport (ESCRT), where it is degraded by the vacuolar protease Pep4. Cells lacking functional AP-3 have decreased levels of polyP, demonstrating that membrane localization of Vtc5 is required for its VTC stimulatory activity *in vivo.* Our work provides insight into the molecular trafficking of a critical regulator of polyP metabolism in yeast. We speculate that AP-3 may also be responsible for the delivery of polyP regulatory proteins to platelet dense granules in higher eukaryotes.

## INTRODUCTION

Polyphosphates (polyP) are chains of inorganic phosphates found in all cell types studied to date. PolyP chains are variable in size, ranging from 3 units to thousands of units in length, and are linked together via high-energy phosphoanhydride bonds (1). While they were once dismissed as “molecular fossils,” recent work suggests that polyP plays critical roles in diverse processes across both prokaryotic and eukaryotic organisms, including bacterial virulence, infection control, blood coagulation, protein folding, and diverse aspects of cell signaling (2–7). As such, polyP chains have gained significant interest as a potential target for therapeutics in a wide variety of pathologies. In contrast to bacteria and fungi, the enzymes that synthesize polyP chains in higher eukaryotic cells are largely unknown. There are no clear homologs of either prokaryotic or fungal polyP synthetases in mammals. Recent work by the Abramov group suggests that the mammalian mitochondrial F_o_F_1_ ATPase has polyP synthesis capabilities (8), but the overall contribution of this enzyme to total cellular pools of polyP remains to be tested.

One model organism used to study polyP at a foundational level is the budding yeast Saccharomyces cerevisiae. Here, polyP is present in high concentrations (>200 mM), and the enzymes responsible for its metabolism have been identified (9–11). In yeast, polyP is synthesized by the vacuolar transporter chaperone (VTC) complex. The minimal (core) VTC complex is composed of 3 subunits: Vtc1, Vtc2, or Vtc3, and the catalytic subunit Vtc4 (9, 12). The Vtc3 subunit mostly localizes to the vacuole membrane alongside Vtc1 and Vtc4 (12). On the other hand, the Vtc2 subunit is found at the endoplasmic reticulum and/or cell periphery and relocalizes to the vacuole membrane under phosphate-limited conditions (12). PolyP synthesis by VTC requires simultaneous translocation of polyP into the vacuole lumen (13), where it makes up over 10% of the dry weight of the cell (10). PolyP is also found in lower concentrations in the cytoplasm, plasma membrane, mitochondria, and nucleus, although observed concentrations vary by method of analysis (14). In addition to being the only known polyP synthetase in yeast, the VTC complex has been demonstrated or suggested to play a role in a myriad of cellular activities, such as the stability of vacuolar V-ATPase subunits, vacuole fusion, and microautophagy (15–17). PolyP itself has also been implicated in the regulation of pH balance (18), ion homeostasis (19–21), and phosphate metabolism (18, 22–24).

**FIG 1:**
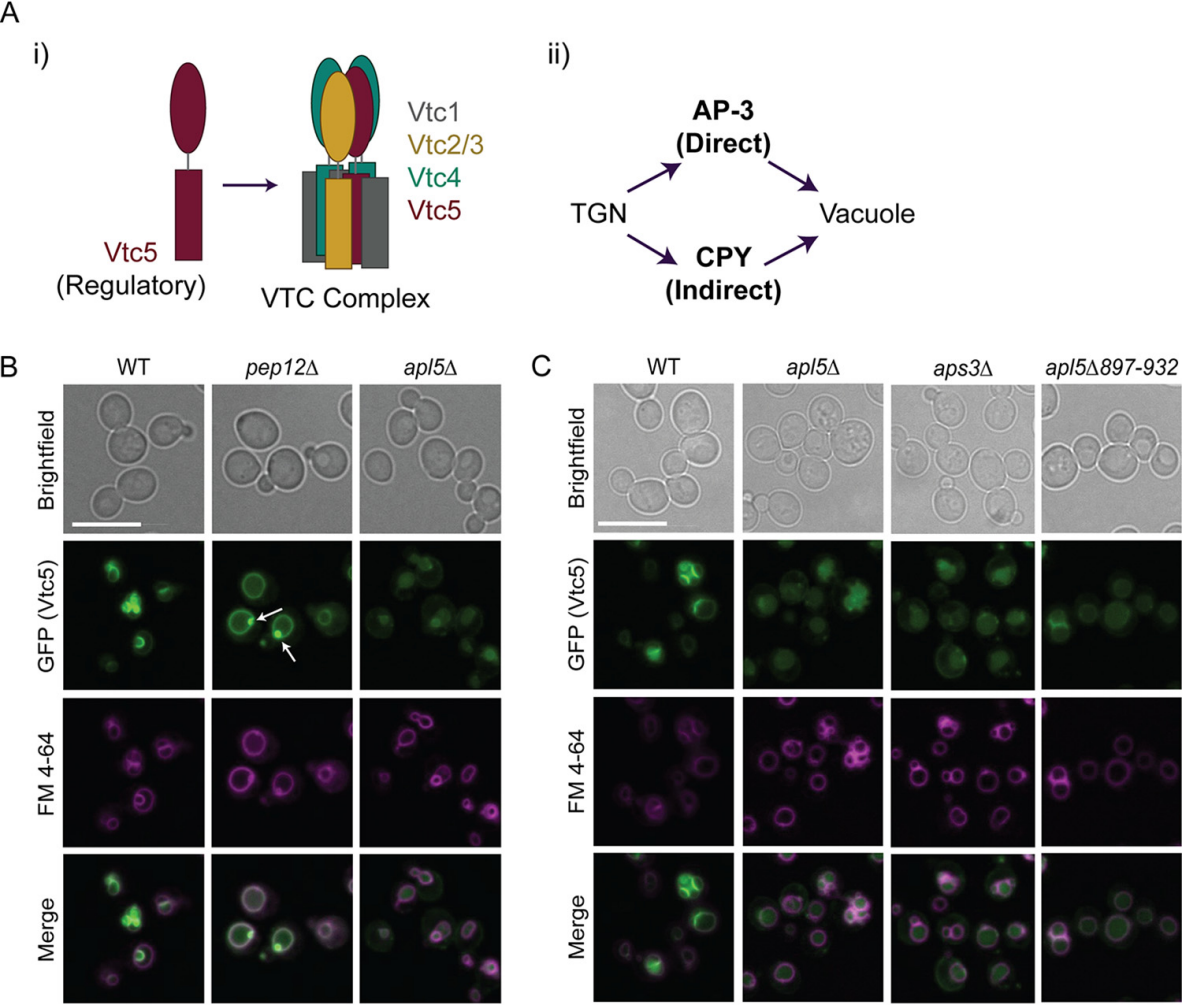
GFP-Vtc5 is localized to the vacuole membrane via the conserved AP-3 pathway. (A) Schematic of the proposed VTC complex subunit organization (i) and a simplified schematic of protein transport to the vacuole in S. cerevisiae (ii). Organization of the VTC complex was proposed by Gerasimaitė et al. (9). (B) The indicated strains were grown in YPD prior to incubation with FM 4-64, which marks the vacuole membrane, for 2 h. Cells were then washed with fresh YPD for 30 min, transferred to synthetic medium, and imaged. Live-cell fluorescence microscopy was performed using a Leica DMI 6000 microscope at 63× with oil immersion. For AP-3 mutants, green (GFP-Vtc5) images were taken at longer exposure times to account for differences in signal intensity. Images were processed in FIJI. Bar, 10 μm. Puncta are indicated by white arrows. (C) The indicated strains were processed as described for panel B. Maximum intensities for individual panels are adjusted to account for variations in intensity of original images. Also see Fig. S1 to S3.

Recent work identified several exciting aspects of VTC regulation. First, activity of the complex is promoted by binding of inositol pyrophosphate InsP7 to the SPX domains of VTC proteins (25, 26). As such, loss of Kcs1, which catalyzes the formation of InsP7, drastically reduces polyP levels (27, 28). Although canonical (serine/threonine) phosphorylation and lysine ubiquitylation sites have been identified on multiple VTC subunits (29–31), their functions are currently unknown. Recently, a new VTC regulatory subunit termed Vtc5 was identified (22) (Fig. 1A, left). Vtc5 localizes exclusively to the vacuole membrane and interacts with the VTC complex to increase the rate of polyP production (22). PolyP levels in *vtc5*Δ mutants are reduced to 20% of those of wild-type cells (22). The mechanism by which Vtc5 exerts its positive effects on VTC is unknown, although it appears to function independently of effects imparted by inositol pyrophosphates (22). Given the importance of Vtc5 as a regulator of the core VTC complex, we sought to identify the pathways that are responsible for localizing Vtc5 to the vacuole membrane.

10.1128/mBio.00994-21.1 REVISED FIG S1 Polyphosphorylation of Apl5 does not impact protein transport. (A) The AP-3 complex is a conserved heterotetramer which includes two large subunits (Apl6/AP3β1 and Apl5/AP3δ1), one medium subunit (Apm3/AP3μ1), and one small subunit (Aps3/AP3σ1). (B) Mutating lysines to arginines (K to R) within Apl5’s PASK cluster causes a collapse in the electrophoretic shift on NuPAGE, indicating a loss of polyphosphorylation. Proteins from the indicated strains were extracted using a TCA protein extraction protocol, electrophoresed on a 4 to 12% NuPAGE gel, and transferred to a PVDF membrane. The membrane was developed with autoradiography film after immunoblotting with an anti-GFP antibody to detect C-terminal Apl5-GFP fusion alleles. Anti-Rts1 served as a positive control for polyphosphorylation. Ponceau S stain was used as a loading control. (C) GFP-Vtc5 is localized to the vacuole membrane in Apl5_PASK_K-R mutants. Cells were grown in YPD prior to incubation with FM 4-64, which marks the vacuole membrane, for 2 h. Cells were then washed with fresh YPD for 30 min, transferred to synthetic medium, and imaged. Live-cell fluorescence microscopy was performed using a Leica DMI 6000 microscope at 63× with oil immersion. In the AP-3-null mutant (*apl5*Δ), green (GFP-Vtc5) images were taken at longer exposure times to account for differences in signal intensity. Images were processed in FIJI. Maximum intensities for individual panels are adjusted to account for variations in intensity of original images. Bar, 10 μm. (D) Apl5_PASK_K-R does not impact maturation of AP-3 target Pho8. Proteins were extracted from the indicated strains using a TCA protein extraction protocol, electrophoresed on a 12% SDS-PAGE gel, and transferred to a PVDF membrane. The membrane was developed with autoradiography film after immunoblotting with an anti-Pho8 antibody. Ponceau S stain is used as a loading control. mPho8, mature Pho8; sPho8, soluble Pho8. Download REVISED FIG S1, TIF file, 2.3 MB [ORIGINAL FIG S1, TIF file, 2.7 MB]. Copyright © 2021 Bentley-DeSousa and Downey. 2021 Bentley-DeSousa and Downey. https://creativecommons.org/licenses/by/4.0/ This content is distributed under the terms of the Creative Commons Attribution 4.0 International license.

Membrane proteins are synthesized by ribosomes at the endoplasmic reticulum and transported to the trans-Golgi network (TGN) prior to being sorted to their final destination (32). Vacuolar proteins are sorted by two well-established protein transport pathways, the CPY (indirect; carboxypeptidase Y) and AP-3 (direct; adaptor protein complex 3) transport pathways (Fig. 1A, right). The CPY pathway transports cargoes to the vacuole in an indirect fashion using the endosomal system as an intermediate path. In this pathway, cargoes localize to endosomes prior to fusion with the vacuole membrane for delivery (32). In contrast, AP-3 cargoes are selected at the TGN, where the complex buds from the TGN with its cargoes to create AP-3 coated vesicles. These vesicles are transported directly to the vacuole through the cytoplasm, and AP-3 docks at the vacuole membrane to release its cargoes (Fig. 1A, right) (32). There are links between the AP-3 complex and polyP storage in higher eukaryotes. In humans, mutations in AP-3 are associated with a rare disorder called Hermansky-Pudlak syndrome (33–35). Hermansky-Pudlak syndrome patients lack the ability to synthesize lysosome-related organelles in diverse cell types throughout the body, which results in broad phenotypes that include albinism, visual impairment, and bleeding problems (35). Notably, this includes the inability to generate dense granules in platelets, where polyP is usually stored in its highest concentrations (130 mM) (36). PolyP promotes blood clotting by acting on proteins involved in the coagulation cascade, including factor XII (37), factor XI (38), factor V (39), and thrombin (40). PolyP addition re-establishes clotting stimulatory activity of platelets derived from Hermansky-Pudlak syndrome patients (41).

In this study, we describe a role for the yeast AP-3 complex in localizing Vtc5 to the vacuole membrane. In AP-3 mutants, Vtc5 is rerouted to the vacuole lumen by the endosomal sorting complex required for transport (ESCRT), where it is degraded by the vacuolar protease Pep4. Mislocalization of Vtc5 in AP-3 mutants is accompanied by a decrease in VTC protein levels and decreased levels of polyP. Overall, our study explains the polyP accumulation defects in AP-3 mutants and provides novel insights into the regulation of the VTC polyP synthetase in budding yeast.

## RESULTS

### Vtc5 localization to the vacuole membrane is disrupted in AP-3 mutants.

The CPY and AP-3 pathways constitute the two major routes of protein transport to the vacuole from the TGN (Fig. 1A). To test which of these pathways is responsible for Vtc5 localization to the vacuole membrane, we used live-cell fluorescence microscopy to analyze green fluorescent protein (GFP)-Vtc5 localization in AP-3 and CPY pathway mutants. Notably, all N-terminal GFP-Vtc fusions used in this work are functional, with the GFP tag facing the cytoplasmic side of the vacuole membrane to ensure that the tag is not degraded within the vacuole lumen (12, 22). These GFP fusions are expressed from constitutive promoters integrated at endogenous *VTC* loci (see Materials and Methods). In wild-type cells, GFP-Vtc5 (green) localized exclusively to the vacuole membrane, as demonstrated by colocalization with FM 4-64 (magenta), a dye which labels the vacuole membrane (Fig. 1B). CPY mutants (*pep12*Δ) also showed GFP-Vtc5 accumulation on the vacuole membrane, but this localization was accompanied by the appearance of puncta at the surface of some vacuoles (Fig. 1B). These puncta may signify partially defective transport through this pathway (42). In contrast, GFP-Vtc5 was clearly mislocalized to the vacuole lumen in AP-3 mutant (*apl5*Δ) cells (Fig. 1B). AP-3 is highly conserved from yeast to humans. It consists of a heterotetramer of four protein subunits: two large subunits (Apl6/AP3β1 and Apl5/AP3δ1), one medium subunit (Apm3/AP3μ1), and one small subunit (Aps3/AP3σ1) (see Fig. S1A in the supplemental material) (43). Deletion of genes encoding any one of the four AP-3 subunits results in defects in AP-3 cargo transport (44). Therefore, to corroborate our results with *apl5*Δ, we examined GFP-Vtc5 localization in *aps3*Δ cells and again observed its mislocalization to the vacuole lumen in this mutant (Fig. 1C). While the presence of GFP-Vtc5 on the vacuole membrane to appears to depend on AP-3, we cannot rule out the possibility that the CPY pathway may also contribute to its delivery under some circumstances.

Intriguingly, polyP chains can be covalently attached to protein targets as a posttranslational modification termed polyphosphorylation (27, 45). Polyphosphorylation is the nonenzymatic addition of polyP chains onto lysine residues, principally within poly-acidic, serine, and lysine (PASK)-rich clusters (27, 46). We previously reported that Apl5 is polyphosphorylated in its C-terminal PASK cluster (amino acids 897 to 932) (47). Therefore, to test if polyphosphorylation impacts Apl5’s role in localizing GFP-Vtc5 to the vacuole membrane, we first deleted the PASK cluster in its entirety. Deletion of Apl5’s PASK cluster (*apl5*Δ897–932) resulted in mislocalization of GFP-Vtc5 to the vacuole lumen, similar to what we observed in AP-3-null mutants (Fig. 1C). To define the contribution of polyphosphorylation more specifically, we generated a strain wherein endogenous Apl5 is expressed with 13 lysine-to-arginine (K-R) amino acid substitutions in its PASK cluster. Polyphosphorylation results in an electrophoretic shift of target proteins separated on bis-Tris NuPAGE gels (27, 48), and this is currently the only method described for evaluation of this new modification. As demonstrated by NuPAGE analysis, the resulting mutant (Apl5_PASK_K-R) was unable to undergo polyphosphorylation (Fig. S1B). However, this did not impact GFP-Vtc5 localization (Fig. S1C), nor did it mimic other known *apl5*Δ phenotypes, such as defects in the maturation of AP-3 target Pho8, a vacuolar alkaline phosphatase (49) (Fig. S1D), or enhanced sensitivity to nickel chloride (50) or rapamycin (51) (Fig. S2A and B). We note that the PASK cluster lies within Apl5’s Vps41 binding domain (52). Vps41 is a member of the homotypic fusion and protein sorting (HOPS) complex required for vesicle docking at the vacuole and delivery of AP-3 cargoes (52, 53). As such, the role of the Apl5 PASK in GFP-Vtc5 delivery may stem from disruption of this interaction rather than a defect in polyphosphorylation. Interestingly, the Apl5 C-terminal PASK deletion mutant (*apl5*Δ897-932) showed defects in maturation of Pho8, but it did not result in sensitivity to nickel chloride or rapamycin (Fig. S1D and S2A and B). This separation-of-function mutant may serve as a useful tool to dissect AP-3’s role underlying these distinct phenotypes.

10.1128/mBio.00994-21.2 REVISED FIG S2 Deletion of the Apl5 PASK cluster does not impact sensitivity to rapamycin or nickel chloride. (A) Functional AP-3 is required for growth in the presence of 0.003 μM rapamycin. Cells were grown in YPD to log phase and diluted to an OD_600_ of 0.1 prior to 5-fold serial dilutions in H_2_O. Four microliters of each dilution was spotted on the indicated medium prior to incubation at 30°C for 2 to 4 days. Images were taken on a Bio-Rad ChemiDoc system. DMSO was used as a control for rapamycin treatment. (B) Functional AP-3 is required for growth in the presence of 0.75 mM NiCl_2_. A YPD plate was used as a control. The method was the same as for panel A. Download REVISED FIG S2, TIF file, 2.2 MB [ORIGINAL FIG S2, TIF file, 2.3 MB]. Copyright © 2021 Bentley-DeSousa and Downey. 2021 Bentley-DeSousa and Downey. https://creativecommons.org/licenses/by/4.0/ This content is distributed under the terms of the Creative Commons Attribution 4.0 International license.

We next sought to test whether vacuolar localization of additional VTC subunits was also impacted by *APL5* deletion. We observed no concrete difference in the localization of GFP-Vtc3 in wild-type cells versus *apl5*Δ mutants (Fig. S3A). GFP-Vtc4 showed an intermediate phenotype, with increased localization to the cytoplasm in *apl5*Δ, with some protein also remaining at the vacuole membrane (Fig. S3B). These data suggest that although the AP-3 pathway is responsible for the proper vacuolar localization of GFP-Vtc5, other pathways may contribute to the localization of core VTC subunits. Given the importance of Vtc5 as a regulator of VTC activity and the clear disruption of its localization in AP-3 mutants, we focused on the transport of this subunit.

10.1128/mBio.00994-21.3 REVISED FIG S3 Partial disruption of GFP-Vtc3 and GFP-Vtc4 localization in AP-3 mutants. (A) The indicated strains were grown in YPD medium prior to incubation with FM 4-64, which marks the vacuole membrane, for 2 h. Cells were then washed with fresh YPD for 30 min, transferred to synthetic medium, and imaged. Live-cell fluorescence microscopy was performed using a Zeiss AxioObserver 7 at 63× with oil immersion. Images were processed in FIJI. (B) The indicated strains were processed as for panel A. Bar, 10 μm. Download REVISED FIG S3, TIF file, 2.3 MB [ORIGINAL FIG S3, TIF file, 2.2 MB]. Copyright © 2021 Bentley-DeSousa and Downey. 2021 Bentley-DeSousa and Downey. https://creativecommons.org/licenses/by/4.0/ This content is distributed under the terms of the Creative Commons Attribution 4.0 International license.

### GFP-Vtc subunits are degraded in cells lacking functional AP-3.

**FIG 2:**
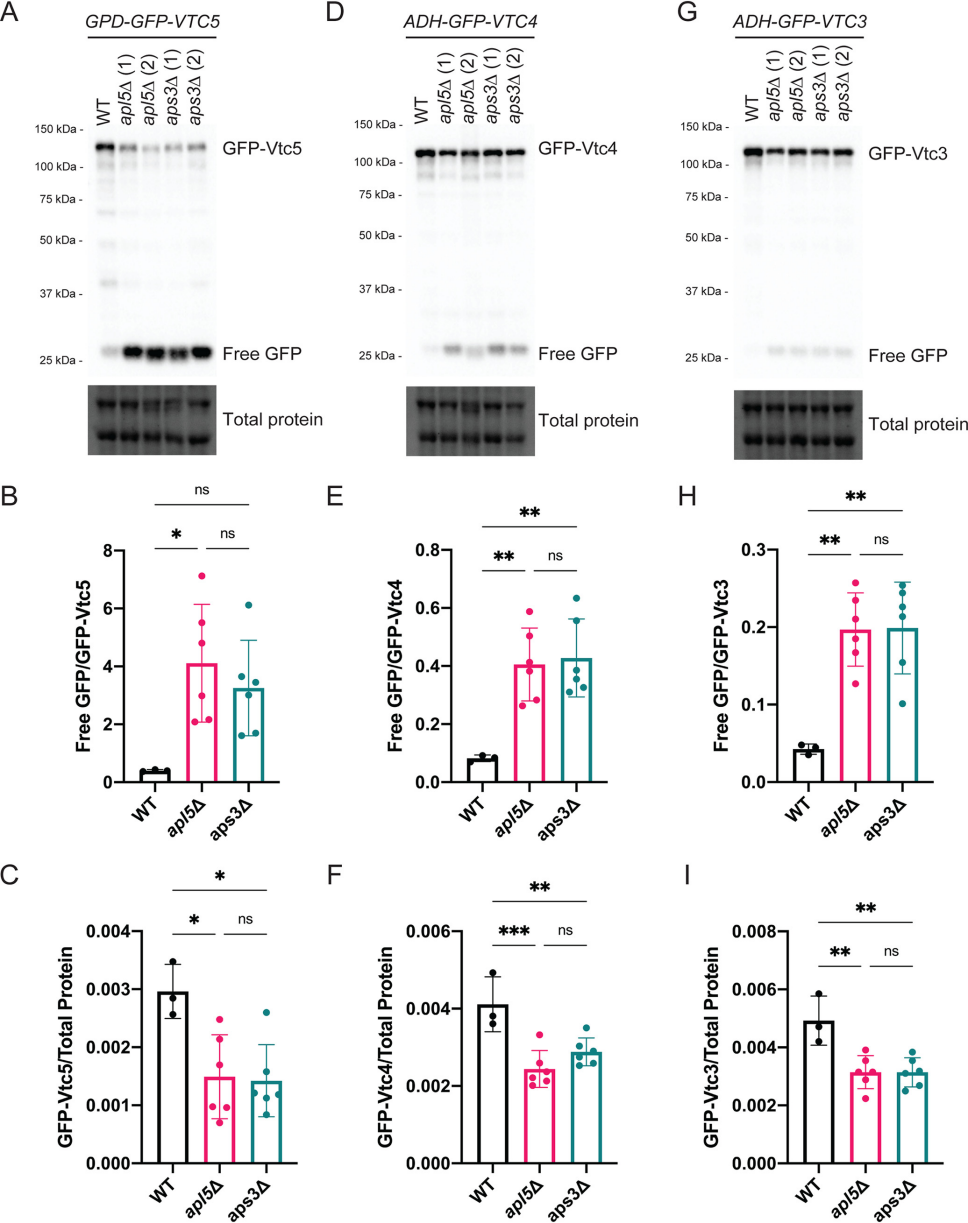
AP-3 mutation causes degradation of GFP-Vtc proteins. (A) Upon AP-3 mutation (*apl5*Δ and *aps3*Δ), full-length GFP-Vtc5 protein levels are reduced with a concomitant increase in free GFP. Proteins were extracted from the indicated strains using a TCA protein extraction protocol, separated on a 10% Bio-Rad TGX Stain-Free FastCast acrylamide gel, and transferred to a nitrocellulose membrane. The membrane was imaged on a Bio-Rad ChemiDoc system after immunoblotting with anti-GFP. Total protein was imaged as a loading control. (B and C) Quantification of free-GFP/GFP-Vtc5 ratios and full-length GFP-Vtc5 levels. Quantifications were done using Bio-Rad ImageLab software, and graphs were created using Prism GraphPad software. One-way ANOVA were performed with Tukey *post hoc* tests. *, *P < *0.05; **, *P < *0.01; ***, *P* < 0.001; ns, not significant. Error bars represent standard deviations of the mean. *n* = 3 for the wild type (WT) and *n* = 6 for AP-3 mutants. (D to F) In AP-3 mutants, GFP-Vtc4-expressing strains accumulate free GFP at the expense of full-length protein. The methods were the same as for panels A to C. (G to I) In AP-3 mutants, GFP-Vtc3-expressing strains accumulate free GFP at the expense of full-length protein. The methods were the same as for panels A to C.

We next used Western blotting to gain insight into the fate of mislocalized GFP-Vtc5. Relative to wild-type controls, *apl5*Δ and *aps3*Δ cells showed a striking accumulation of free GFP, which is known to be resistant to degradation (Fig. 2A and B) (54). This was not the result of increased protein expression, as full-length GFP-Vtc5 was reduced in these mutants (Fig. 2A and C). This pattern was observed previously for other proteins mislocalized to the vacuole lumen and is attributed to cargo degradation (55, 56). We also tested if GFP-Vtc4 and GFP-Vtc3 levels were similarly affected. Both GFP-Vtc4 and GFP-Vtc3 also accumulated free GFP at the expense of decreased full-length fusions, although the effect was not as dramatic as that observed for GFP-Vtc5 (Fig. 2D to I). Previous work from the Mayer group showed that *vtc5*Δ cells have decreased levels of core VTC subunits at the vacuole membrane (22). Degradation of GFP-Vtc3 and GFP-Vtc4 and partial mislocalization of GFP-Vtc4 in *apl5*Δ cells are consistent with a model wherein mislocalized Vtc5 is largely nonfunctional in the absence of AP-3. However, we cannot exclude the possibility that these molecular phenotypes stem in part from defects in the transport of other AP-3 cargoes (see Discussion).

### Mislocalized Vtc5 is rerouted to the vacuole lumen by the ESCRT pathway.

**FIG 3:**
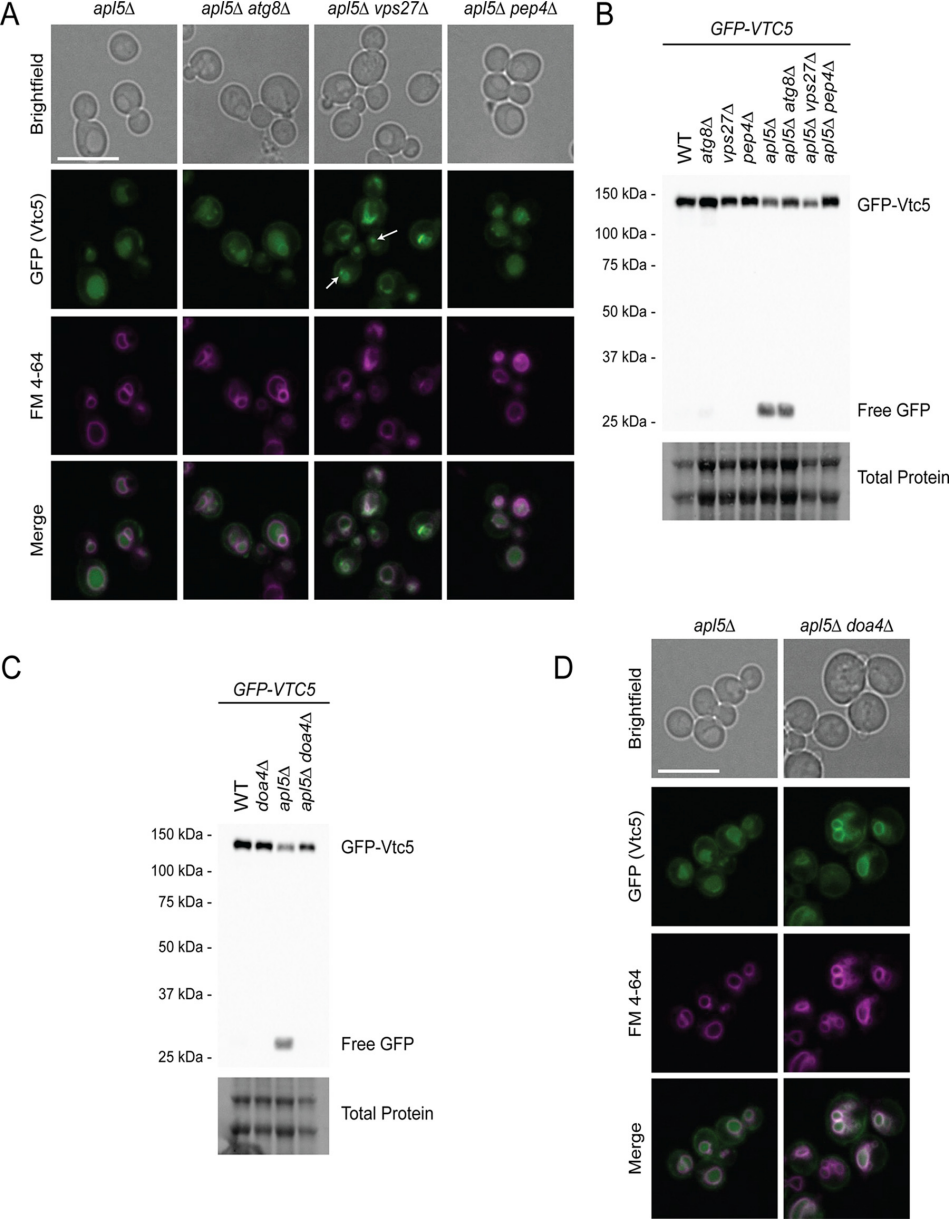
Degradation of mislocalized GFP-Vtc5 depends on ESCRT and the Pep4 protease. (A) The mislocalization of GFP-Vtc5 upon AP-3 mutation is mediated by the ESCRT pathway. Cells were grown in YPD prior to incubation with FM4-64, which marks the vacuole membrane, for 2 h. Cells were then washed with fresh YPD for 30 min, transferred to synthetic medium, and imaged. Live-cell fluorescence microscopy was performed using a Leica DMI 6000 at 63× with oil immersion. Images were processed in FIJI. Bar, 10 μm. White arrows indicate potential MVBs at the vacuole. For *apl5*Δ and *apl5*Δ *atg8*Δ strains, maximum intensities for the FM 4-64 channel were set lower than for other strains to accommodate differences in FM 4-64 staining. (B) Free GFP accumulation in GFP-Vtc5 strains, mediated by AP-3 mutation, is reversed by ESCRT (*vps27*Δ) and *pep4*Δ mutation. Proteins from the indicated strains were extracted using a TCA protein extraction protocol, separated on a 10% Bio-Rad TGX Stain-Free FastCast acrylamide gel, and transferred to a nitrocellulose membrane. The membrane was imaged for total protein and probed using an anti-GFP antibody to detect GFP-Vtc5 protein using a Bio-Rad ChemiDoc. (C) GFP-Vtc5 degradation and free GFP accumulation is reversed by *DOA4* deletion. The method was the same as for panel B. (D) Deletion of *DOA4* rescues localization of GFP-Vtc5 in *apl5*Δ mutants. The method was the same as for panel A. Also see Fig. S4.

We next investigated which pathways are responsible for localizing GFP-Vtc5 to the vacuole lumen in the absence of functional AP-3, with a focus on the autophagy and ESCRT pathways. Autophagy is a process whereby cytoplasmic material becomes sequestered in vesicles that fuse to the vacuole to deliver their contents for degradation (57). The ESCRT pathway is responsible for detecting ubiquitylated transmembrane proteins to sort them into multivesicular bodies (MVBs) in the endocytic pathway for delivery to the vacuole (58). Disruption of ESCRT (*vps27*Δ) but not autophagy (*atg8*Δ) in AP-3 mutants resulted in a loss of GFP signal from the vacuole lumen (Fig. 3A). In contrast, neither pathway impacted GFP-Vtc5 localization in wild-type cells (Fig. S4A). When ESCRT subunits are mutated, the complex becomes defective for proper MVB biogenesis and fusion at the vacuole membrane (58). Indeed, in *apl5*Δ *vps27*Δ double mutants, GFP-Vtc5 appeared to accumulate in FM 4-64-labeled vesicles around the vacuole membrane (Fig. 3A). Deletion of *VPS27* also resulted in reversal of free GFP accumulation in Western blots (Fig. 3B). The same molecular phenotype was observed across multiple ESCRT mutants (ESCRT-0, -I, -II, and -III) (Fig. S4B).

10.1128/mBio.00994-21.4 REVISED FIG S4 ESCRT is responsible for GFP-Vtc5 degradation only when AP-3 is nonfunctional. (A) In wild-type cells, GFP-Vtc5 remains localized to the vacuole membrane in autophagy-deficient (*atg8*Δ), ESCRT-deficient (*vps27*Δ), and protease-deficient (*pep4*Δ) cells. The indicated strains were grown in YPD prior to incubation with FM 4-64, which marks the vacuole membrane, for 2 h. Cells were then washed with fresh YPD for 30 min, transferred to synthetic medium, and imaged. Live-cell fluorescence microscopy was performed using a Leica DMI 6000 microscope at 63× with oil immersion. Images were processed in FIJI. Bar, 10 μm. (B) Mutations in ESCRT-0 (*vps27*Δ), ESCRT-I (*vps23*Δ), ESCRT-II (*vps36*Δ), and ESCRT-III (*vps20*Δ) reverse free GFP accumulation in AP-3 mutants expressing GFP-Vtc5. Proteins were extracted using a TCA protein extraction protocol, electrophoresed on a 10% Bio-Rad TGX Stain-Free FastCast acrylamide gel, and transferred to a nitrocellulose membrane. The membrane was imaged using a Bio-Rad ChemiDoc system after immunoblotting with an anti-GFP antibody to detect GFP-Vtc5. Total protein was imaged as a loading control. (C) Deletion of *DOA4* does not impact wild-type GFP-Vtc5 localization. The method was the same as for panel A. For *doa4*Δ, maximum intensities for the GFP channel were set lower than for the WT to accommodate different intensities of original images. Download REVISED FIG S4, TIF file, 2.7 MB [ORIGINAL FIG S4, TIF file, 3.0 MB]. Copyright © 2021 Bentley-DeSousa and Downey. 2021 Bentley-DeSousa and Downey. https://creativecommons.org/licenses/by/4.0/ This content is distributed under the terms of the Creative Commons Attribution 4.0 International license.

The ESCRT pathway recognizes cargoes that have been ubiquitylated by E3 ubiquitin ligases (58, 59). These cargoes are then transported to the vacuole via MVBs that fuse with the vacuole membrane. As one of the last steps in this process, the deubiquitinase Doa4 removes and recycles ubiquitin moieties from ESCRT-targeted proteins upon cargo delivery to the vacuole (58, 59). Mutation of *DOA4* alone had no impact on GFP-Vtc5 processing or localization (Fig. 3C and Fig. S4C). In AP-3 mutants, however, *doa4*Δ prevented the accumulation free GFP, mirroring what was observed for ESCRT mutants (Fig. 3C). Moreover, *doa4*Δ also restored robust GFP-Vtc5 localization to the vacuole membrane (Fig. 3D). Taking these results together, we conclude that Doa4 is a key player in localizing Vtc5 to the vacuole lumen in the absence of AP-3. Finally, when proteins accumulate in the lumen, they become accessible to vacuole proteases (60). Deletion of the major vacuolar protease Pep4 reversed the accumulation of free GFP in AP-3 mutants (Fig. 3B), without impacting total GFP accumulation in the vacuole lumen (Fig. 3A), suggesting that Pep4 is a major protease responsible for GFP-Vtc5 degradation.

### The AP-3 complex is required for maintenance of wild-type polyP levels.

**FIG 4:**
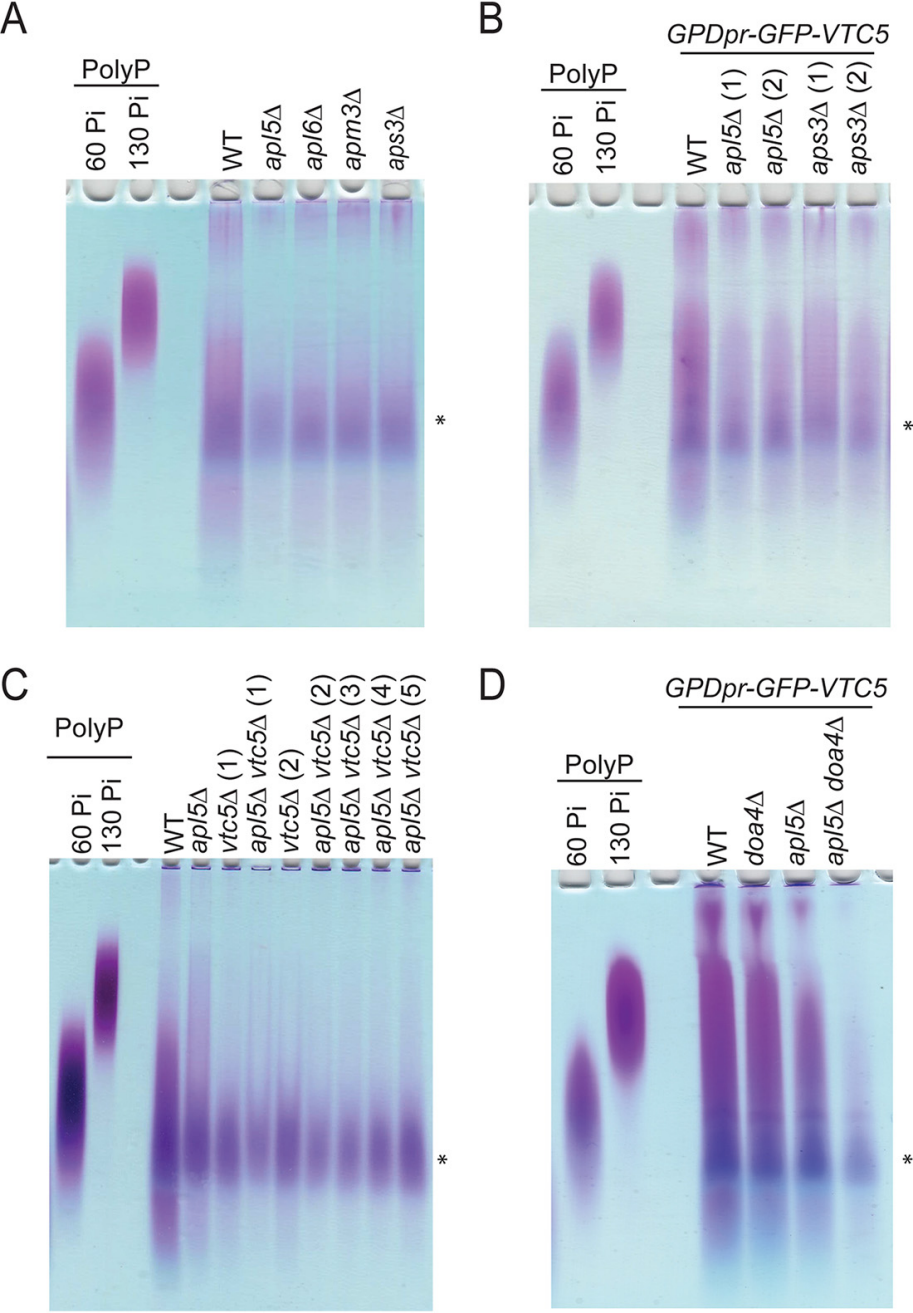
Functional AP-3 is required for the maintenance of polyP levels. (A) An AP-3 subunit mutant causes a reduction in polyP levels. There is a loss of polyP when each of the four AP-3 subunits (*apl5*Δ, *apl6*Δ, *apm3*Δ, and *aps3*Δ) is mutated. PolyP was extracted as described in Materials and Methods. After extraction, samples were mixed with polyP loading dye and separated on a 15.8% TBE-urea acrylamide gel. The gel was incubated in fixing solution with toluidine blue for 15 min prior to destaining and imaging. PolyP with chain lengths of 60 and 130 P_i_ units are included as standards. (B) AP-3 mutants (*apl5Δ and aps3*Δ) cause a reduction in polyP levels when GFP-Vtc5 is expressed under the control of a *GPD1* promoter. The method was the same as for panel A. (C) Mutating AP-3 (*apl5*Δ) and *vtc5*Δ results in a largely epistatic decrease in polyP levels. The method was the same as for panel A. (D) Although *doa4*Δ is capable of rescuing GFP-Vtc5 localization and protein degradation when AP-3 is mutated (Fig. 3C and D), it is incapable of restoring polyP levels. The method was the same as for panel A. The asterisks indicate a background contaminant in extractions. Numbers indicate independent strains.

Finally, we tested the contribution of AP-3 to polyP homeostasis. Deletion of AP-3 subunits resulted in a clear decrease in polyP levels (Fig. 4A). This finding is consistent with previous data wherein AP-3 mutants were found to be important for polyP accumulation in a large-scale screen (61). We observed a similar result in our strains used for prior analyses where GFP-Vtc5 is expressed under the control of a constitutive *GPD1* promoter (Fig. 4B). Together with our previous results, these data suggest that correct localization of Vtc5 to the vacuole membrane by the AP-3 complex is important for its function. Notably, AP-3 mutants still have more polyP than cells lacking Vtc5 altogether (*vtc5*Δ) (Fig. 4C). This observation could be explained by residual localization of Vtc5 to the vacuole membrane in AP-3 mutants. We note that the decrease in polyP observed in *apl5*Δ mutants is largely epistatic with *vtc5*Δ, consistent with the notion that they function within the same pathway (Fig. 4C). Interestingly, deletion of *DOA4*, which rescues GFP-Vtc5 processing and localization to the vacuole membrane, was unable to reverse the decreased polyP levels in *apl5*Δ mutants (Fig. 4D). Thus, proper function of GFP-Vtc5 requires localization to the vacuole membrane, specifically through the AP-3 pathway.

## DISCUSSION

### AP-3 regulation of Vtc5 localization.

Interest in polyP research has experienced a resurgence in recent years on the heels of the discovery of exciting connections between polyP and diverse aspects of cell signaling and protein homeostasis. With the key players involved in mammalian polyP metabolism largely uncharacterized, model systems have proved essential to our understanding of polyP dynamics and function. In S. cerevisiae, polyP is synthesized by the vacuole-bound VTC complex and stored at high concentrations in the vacuole (13). The activity of the core VTC complex (consisting of Vtc1, Vtc2 or Vtc3, and Vtc4) is increased dramatically by the Vtc5 subunit (22). This is the only protein known to act directly on the VTC complex to stimulate polyP production. Our study supports a model where Vtc5 localization to the vacuole membrane depends on the evolutionarily conserved AP-3 complex. This finding provides insight into the regulation of the VTC complex and identifies Vtc5 as a cargo whose mislocalization underlies polyP accumulation defects observed in AP-3 mutants.

**FIG 5:**
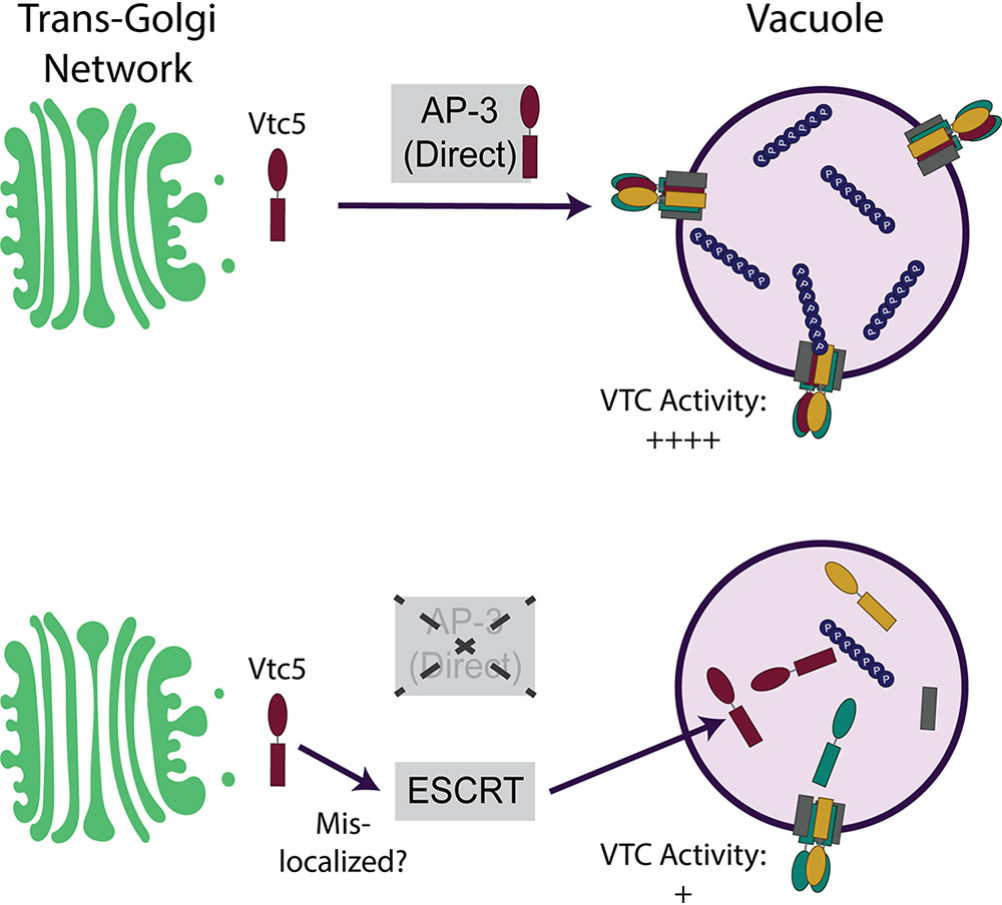
GFP-Vtc5 is localized to the vacuole membrane via the AP-3 complex. In the absence of AP-3, mislocalized GFP-Vtc5 could be localized at the plasma membrane or elsewhere in the cell prior to recognition by ESCRT and delivery to the vacuole lumen for Pep4-dependent degradation. However, we cannot rule out the possibility that Vtc5 is incorrectly inserted at the vacuole membrane prior to ESCRT-dependent internalization. See the text for details.

We propose that in wild-type cells (Fig. 5), Vtc5 is sorted into AP-3-coated vesicles at the TGN and transported directly to the vacuole. The AP-3 complex selects protein cargoes by tyrosine-based motifs (YXXØ, where X represents any amino acid and Ø is a bulky hydrophobic amino acid) and/or dileucine-based motifs ([D/E]XXXL[L/I], where X represents any amino acid) (62). Notably, Vtc5 has 6 tyrosine-based motifs and 2 dileucine-based motifs, which together could be used as a targeting signal. Alternatively, Vtc5 could be transported in conjunction with other AP-3 cargoes, such as Vam3, which is also required for wild-type levels of polyP accumulation (61). Regardless, it is likely that Vtc5 delivery requires interaction of AP-3 coated vesicles with Vps41 of the HOPS complex, which facilitates docking and release of cargo into the vacuole membrane (52, 63–65). In the absence of AP-3, Vtc5 is mislocalized to the vacuole lumen. Mislocalized Vtc5 (e.g., in *apl5*Δ), is recognized and sorted by the ESCRT pathway into the vacuole lumen via the endosomal system, where it is eventually degraded in a manner dependent on vacuolar protease Pep4 (Fig. 5). Since Pep4 is also required for the activation of vacuolar proteases Prb1 and Prc1 (66, 67), it is possible that these also play a role in Vtc5 degradation in the vacuole lumen.

We found that degradation of Vtc5 in AP-3 mutants requires the Doa4 deubiquitinase. *DOA4* is required for sorting of many ESCRT-dependent cargoes into the endosomal system (68), and *doa4*Δ mutants have decreased levels of free ubiquitin (69). Therefore, it is very likely that mislocalized Vtc5 is subject to regulation by ubiquitylation. Indeed, several large-scale studies have identified ubiquitylation sites in the middle and C-terminal regions of the protein (70). We speculate that these sites may be targets of the Rsp5 E3 ubiquitin ligase, which has been implicated in the ESCRT-dependent delivery of proteins to the vacuole lumen (71–73). Notably, Vtc5 is also rich in phosphoserines, including those potentially regulated by the Cdk1 (cell cycle) and Nnk1 (nitrogen metabolism) kinases (70). These modifications could impact Vtc5 localization in wild-type or AP-3 mutant cells or may instead be involved in regulating its activity toward the core VTC complex.

### Regulation of the core VTC complex.

In contrast to GFP-Vtc5, we found that localization of GFP-Vtc3 to the vacuole membrane was not impacted by disruption of AP-3 and GFP-Vtc4 was only partially affected. However, deletion of AP-3 subunits still reduced GFP-Vtc3/4 protein levels and the increased appearance of free GFP in Western blots, albeit to a lesser degree than that observed for GFP-Vtc5. We suggest that transport of core VTC subunits occurs through multiple transport routes, although these may function redundantly with AP-3. This type of dual regulation has been documented for other AP-3 cargoes, such as Sna4 (56) and Ypq1 (74). It is also possible that changes observed in core subunits stem from mislocalized Vtc5. There is strong evidence that maximal polyP production requires VTC localization to the vacuole (13). However, polyP has also been detected in variable amounts at the plasma membrane, cytoplasm, mitochondria, and nucleus (14). Whether vacuolar polyP is somehow transported to these compartments or whether it is made by VTC residing locally remains an open question. In the latter case, it is possible that a lack of Vtc5 colocalization with core VTC subunits in these areas ensures that subcellular concentrations of polyP outside the vacuole are kept low. However, it is not clear if Vtc5 localization may change throughout the cell cycle or in response to specific stresses, and this remains an important area for future work. It will also be intriguing to identify pathways that regulate localization of GFP-Vtc2, which is found at the plasma membrane or peripheral endoplasmic reticulum under phosphate-replete conditions, becoming enriched at the vacuole only under conditions of phosphate starvation (9, 12, 15).

### AP-3 impact on polyP levels.

Our work shows that AP-3 mutant cells have reduced levels of polyP. This was true both in a wild-type background and under conditions of Vtc5 overexpression. These findings are consistent with work from Freimoser et al. identifying AP-3-encoding genes in a genome-wide screen for deletion mutations with defects in polyP accumulation (61). Interestingly, deletion of *DOA4* in an AP-3 mutant background rescued both GFP-Vtc5’s localization to the vacuole membrane and GFP-Vtc5 degradation without restoring polyP levels. In fact, polyP levels in *apl5*Δ *doa4*Δ double mutants were lower than in either single mutant. While this may seem counterintuitive, we suggest that Vtc5 is not correctly positioned within the vacuole membrane under these circumstances and is unable to stimulate VTC activity. The reduction in polyP levels seen in AP-3 mutants is not as dramatic as that observed in cells lacking Vtc5 altogether, suggesting either that there is residual localization of Vtc5 to the vacuole membrane or that mislocalized Vtc5 remains competent to stimulate core VTC activity to some degree. Notably, Freimoser et al. identified over 200 additional genes that impact polyP metabolism (61), and some of these could function as direct regulators of Vtc5 or AP-3.

We recently described the Apl5 subunit of AP-3 as a target of lysine polyphosphorylation (47). However, our analysis of mutant Apl5 that cannot be polyphosphorylated suggests that this modification does not impact GFP-Vtc5 delivery to the vacuole or other AP-3 related phenotypes that we tested. Polyphosphorylation of AP-3 may become important under selected stress conditions that remain to be identified. Since polyP has been described as having chaperone activity (4), another possibility is that polyphosphorylation promotes degradation or refolding of a small fraction of AP-3 that is itself mislocalized to the polyP-rich vacuole lumen.

### Conservation of the AP-3 complex and function.

The AP-3 complex is highly conserved in mammalian cells in terms of subunit organization and function. For example, the human homolog of Ypq1, PQLC2, is transported to the vacuole membrane by the AP-3 complex when it is expressed in yeast (74). Notably, mutations in human AP-3 give rise to Hermansky-Pudlak syndrome. Relevant here is the observation that molecular phenotypes of Hermansky-Pudlak syndrome patients include a failure to accumulate polyP in platelet dense granules, a type of lysosome-related organelle conceptually similar to the yeast vacuole (33–35). Based on the conserved role of AP-3 subunits in polyP metabolism, we suggest that identification and characterization of AP-3 cargoes in human cells that accumulate high levels of polyP may provide unique insights into the human polyP synthetases, the identity of which remains a critical open question in the field.

## MATERIALS AND METHODS

### Yeast strains and handling.

Yeast strains were constructed using standard techniques via transformation of PCR products containing selectable markers. For gene deletions, PCR analyses were used to confirm the position of the gene deletion and the absence of a wild-type gene copy. PCR was also used to confirm the correct genomic location of cassettes used for epitope tagging. Genotypes for all strains used in this study are listed in the supplemental tables. For all experiments performed strains were grown in yeast extract-peptone-dextrose (YPD; 2% glucose supplemented with 0.005% adenine and 0.005% tryptophan).

### Apl5 K-R mutagenesis.

A construct containing the lysine (K)-to-arginine (R) mutations within Apl5 PASK cluster was custom created by GenScript and used for the experiments listed in Fig. S1 and S2. The construct was integrated with a GFP tag or FLAG tag at the endogenous *APL5* locus using transformation of overlapping PCR products.

### Electrophoresis and immunoblotting.

Methods for protein extraction were described previously (48) and are summarized here using similar wording for clarity. A BioSpec bead beater was used to lyse cell pellets corresponding to 3 to 6 units of optical density at 600 nm (OD_600_) in the presence of 100 μl of acid-washed beads and 300 μl of 20% trichloroacetic acid (TCA) (Sigma-Aldrich T6399). Two 3-min pulses were used. The supernatant was recovered, and cells were washed with 300 μl of 5% TCA (Sigma-Aldrich T6399). The supernatant from the second wash was combined with the first, and this mixture was clarified by centrifugation at 4°C at 16,000 × *g* for 4 min. The supernatant was removed, and the pellet was resuspended in SDS-PAGE sample buffer (see buffer recipes below) supplemented with 1/10 volume of 1.5 M Tris-HCl, pH 8.8 (Tris base [Fisher BP152-5], hydrochloric acid [Fisher A144-212]), and 1/10 volume 1 M dithiothreitol (DTT) (Bio Basic DB0058). Samples were boiled for 5 min before an additional centrifugation at 4°C at 16,000 × *g* for 4 min. The supernatant was recovered and stored or used immediately for SDS-PAGE or NuPAGE analysis (Thermo Fisher NP0336). Where indicated, Bio-Rad TGX Stain-Free FastCast 10% acrylamide (Bio-Rad 1610183) was used for quantification, with total protein quantified in place of a loading control. Bio-Rad TGX acrylamide gels were transferred to nitrocellulose membranes (Bio-Rad 162-0112), and exposures were obtained using a Bio-Rad ChemiDoc system. Non-TGX 12% SDS-PAGE and NuPAGE gels (Thermo Fisher NP0336) were transferred to polyvinylidene difluoride (PVDF) membranes (Bio-Rad 162-0177), and exposures were obtained using autoradiography film (Harvard Apparatus Canada DV-E3018). In all cases, Luminata Forte enhanced chemiluminescence (ECL) (Fisher Scientific WBLUF0500) was employed for detection. All antibodies used for immunoblotting are described in the supplemental tables.

### Spot tests.

Cells were diluted to an OD_600_ of 0.1, grown for 4 h, and then diluted again to an OD_600_ of 0.1 prior to being serially diluted 5-fold in H_2_O. Four microliters of each dilution was spotted on the indicated medium prior to incubation at 30°C for 2 to 3 days. Plates contained the following chemicals/drugs: 0.028% dimethyl sulfoxide (DMSO) (VWR CA97061-250), 0.75 mM NiCl_2_ (Fisher N54-250), and 0.003 μM rapamycin (Sigma-Aldrich R0395-1MG). Images were taken using a Bio-Rad ChemiDoc system.

### Microscopy.

Live-cell fluorescence imaging was conducted using a Leica DMI 6000 microscope with a Hamamatsu camera using the Volocity 4.3.2 imaging program. Briefly, cells were diluted to an OD_600_ of 0.2 from overnight cultures in YPD. After 3 h of growth at 30°C, FM 4-64 (Thermo Fisher T13320; 1.64 mM stock in DMSO) was added to a final concentration of 1.64 μM for an additional 2 h. Cells were then washed out in YPD for 30 min at room temperature. In instances where there was a difference in protein expression level, exposure times were taken at longer intervals to account for this discrepancy (e.g., Fig. 1). For microscopy whose results are shown in Fig. S3, images were taken on a Zeiss AxioObserver 7 microscope with a Hamamatsu ORCA-Flash LT camera using Zeiss Zen 3.0 Pro Software. All images were taken with oil immersion at 63×. All images were then analyzed in FIJI. Backgrounds were subtracted with a rolling-ball radius of 50 pixels, and images taken with FM 4-64 dye were converted from red to magenta. For microscopy images, Mix/Max values in FIJI were chosen to highlight representative changes in subcellular localization across multiple fields of view.

### Polyphosphate extractions.

Polyphosphate was extracted from yeast pellets containing 8 to 12 OD_600_ units using an adapted protocol from Bru et al. (7, 75). Cells were resuspended in 400 μl of cold LETS buffer (see buffer recipes below). Subsequently, 600 μl of neutral phenol pH 8 (Sigma-Aldrich P4557) and 150 μl of Milli-Q H_2_O were added. Samples were vortexed for 20 s and heated for 5 min at 65°C followed by a 1-min incubation on ice. Six hundred microliters of chloroform (Sigma-Aldrich 472476) was added, and samples were vortexed for 20 s and spun down at room temperature for 2 min at 13,000 × *g*. The top layer was then transferred to a new tube containing 600 μl of chloroform (Sigma-Aldrich 472476), vortexed for 20 s and spun down at room temperature for 2 min at 13,000 × *g*. The top layer was transferred to a new tube and 2 μl of RNase A (10 mg/ml; Thermo Fisher R1253) and 2 μl of DNase I (10 mg/ml; Thermo Fisher AM2222) were added, followed by a 1-h incubation at 37°C. The mixture was transferred to a prechilled tube containing 1 ml 100% ethanol (Commercial Alcohols P006EAAN) and 40 μl of 3 M sodium acetate, pH 5.3 (Sigma-Aldrich S7899). Samples were left at −20°C overnight and then centrifuged for 20 min at 13,000 × *g* at 4°C. The pellet was washed in 500 μl of cold 70% ethanol (Commercial Alcohols P006EAAN) and centrifuged for 5 min at 13,000 × *g* at 4°C. The supernatant was discarded, and the pellet was air dried before resuspension in 20 to 30 μl mH_2_O. Samples were mixed 1:1 with polyP loading dye (see buffer recipes below) and electrophoresed on a 15.8% Tris-borate-EDTA (TBE)–urea acrylamide gel at 100 V for 1 h and 45 min in 1× TBE buffer (see buffer recipes below). The gel was incubated in fixing solution (see buffer recipes below) with toluidine blue for 15 min and then destained in destaining solution. PolyP standards (a gift from T. Shiba) were used to assess polyP chain length.

### Statistical analyses.

For statistical analyses performed on Western blots in Fig. 2, a one-way analysis of variance (ANOVA) was performed with Tukey *post hoc* tests at 95% confidence intervals. Error bars, *P* values, and numbers of biological replicates (*n*) are defined in the relevant figure legends.

### Buffer recipes.

The following buffer recipes are taken from our previous study (48) and are listed here for convenience.

SDS-PAGE running buffer (1× working concentration) consists of 100 ml of 10× 1 liter stock (30.2 g Tris base [Fisher BP152-5], 188 g glycine [Fisher BP381-5], 10 g SDS [Fisher BP166]) and 900 ml double-distilled water (ddH_2_O). Note that SDS-PAGE gels do not resolve polyP shifts.

NuPAGE running buffer (1× working concentration) consists of 50 ml of 20× 1 liter stock (209.2 g MOPS [Sigma M1254], 121.1 g bis-Tris [Sigma B9754], 20 g SDS [Fisher BP166], 12 g EDTA [Sigma ED2SS]), 5 ml of 1 M sodium bisulfite (Fisher S654-500), and 950 ml ddH_2_O.

SDS-PAGE transfer buffer (1× working concentration) consists of 100 ml of 10× 1 liter stock (30.275 g Tris base [Fisher BP152-5], 166.175 glycine [BioBasic GB0235]), 200 ml methanol (Fisher A412P-4), and 700 ml ddH_2_O.

NuPAGE transfer buffer (1× working concentration) consists of 50 ml of 20× 1 liter stock (81.6 g Bicine [Sigma B3876], 104.8 g bis-Tris [Sigma B9754], 6 g EDTA [Sigma ED2SS]), 200 ml methanol (Fisher A412P-4), and 750 ml ddH_2_O.

SDS-PAGE/NuPAGE sample buffer (3× stock) consists of 800 μl of stock (160 mM Tris-HCl [pH 6.8] [Tris base {Fisher BP152-5}, hydrochloric acid {Fisher A144-212}], 6% SDS [wt/vol] [Fisher BP166], 30% glycerol [Fisher BP229-4], 0.004% bromophenol blue [Fisher BP115-25]). For TCA preparations, 3× is supplemented with 100 μl 1 M DTT (BioBasic DB0058) and 100 μl 1.5 M Tris-HCl (pH 8.8) (Tris base [Fisher BP152-5], hydrochloric acid [Fisher A144-212]).

The following buffer recipes are from this current study.

TBE (1× working concentration) consists of 200 ml of a 5× stock (67.5 g Tris base [Fisher BP152-5], 34.37 g boric acid [Fisher BP168-1], and 25 ml 0.5 M EDTA [Sigma-Aldrich 03690]) and 800 ml ddH_2_O.

LETS buffer (1× working concentration) consists of 100 mM LiCl (Fisher L120-500), 10 mM EDTA (Sigma-Aldrich 03690), 10 mM Tris-HCl (pH 7.4) (Tris base [Fisher BP152-5], hydrochloric acid [Fisher A144-212]), and 20% SDS (Thermo Fisher AM9820).

PolyP loading dye (6×) consists of 10 mM Tris-HCl (pH 7) (Tris base [Fisher BP152-5], hydrochloric acid [Fisher A144-212]), 1 mM EDTA (Sigma-Aldrich 03690), 30% glycerol (Fisher BP229-4), and bromophenol blue (Fisher BP115-25).

Toluidine blue fixing solution consists of 25% methanol (Fisher A412P), 5% glycerol (Fisher BP229-4), and 0.05% toluidine blue (Sigma-Aldrich T3260).

Destaining solution consists of 25% methanol (Fisher A412P) and 5% glycerol (Fisher BP229-4).

10.1128/mBio.00994-21.5 TABLE S1 Yeast strains used in this work. Download Table S1, XLSX file, 0.01 MB. Copyright © 2021 Bentley-DeSousa and Downey. 2021 Bentley-DeSousa and Downey. https://creativecommons.org/licenses/by/4.0/ This content is distributed under the terms of the Creative Commons Attribution 4.0 International license.

10.1128/mBio.00994-21.6 TABLE S2 Antibodies used in this work. Download Table S2, XLSX file, 0.01 MB. Copyright © 2021 Bentley-DeSousa and Downey. 2021 Bentley-DeSousa and Downey. https://creativecommons.org/licenses/by/4.0/ This content is distributed under the terms of the Creative Commons Attribution 4.0 International license.
