# Erratum for Bentley-DeSousa and Downey, “Vtc5 Is Localized to the Vacuole Membrane by the Conserved AP-3 Complex To Regulate Polyphosphate Synthesis in Budding Yeast”

**DOI:** 10.1128/mBio.02997-21

**Published:** 2021-11-02

**Authors:** Amanda Bentley-DeSousa, Michael Downey

**Affiliations:** a Department of Cellular and Molecular Medicine, University of Ottawa, Ottawa, Ontario, Canada; b Ottawa Institute of Systems Biology, University of Ottawa, Ottawa, Ontario, Canada

## ERRATUM

Volume 12, no. 5, e00994-21, 2021, https://doi.org/10.1128/mBio.00994-21. In the originally published version, the supplemental figures were out of order. The article has been updated to correct the file order.

