# Supplementary figures and images for: Vtc5 Is Localized to the Vacuole Membrane by the Conserved AP-3 Complex to Regulate Polyphosphate Synthesis in Budding Yeast
Source: mBio. 2021 Sep 21;12(5):e00994-21. doi: 10.1128/mBio.00994-21 (PMC8510523; doi:10.1128/mBio.00994-21)

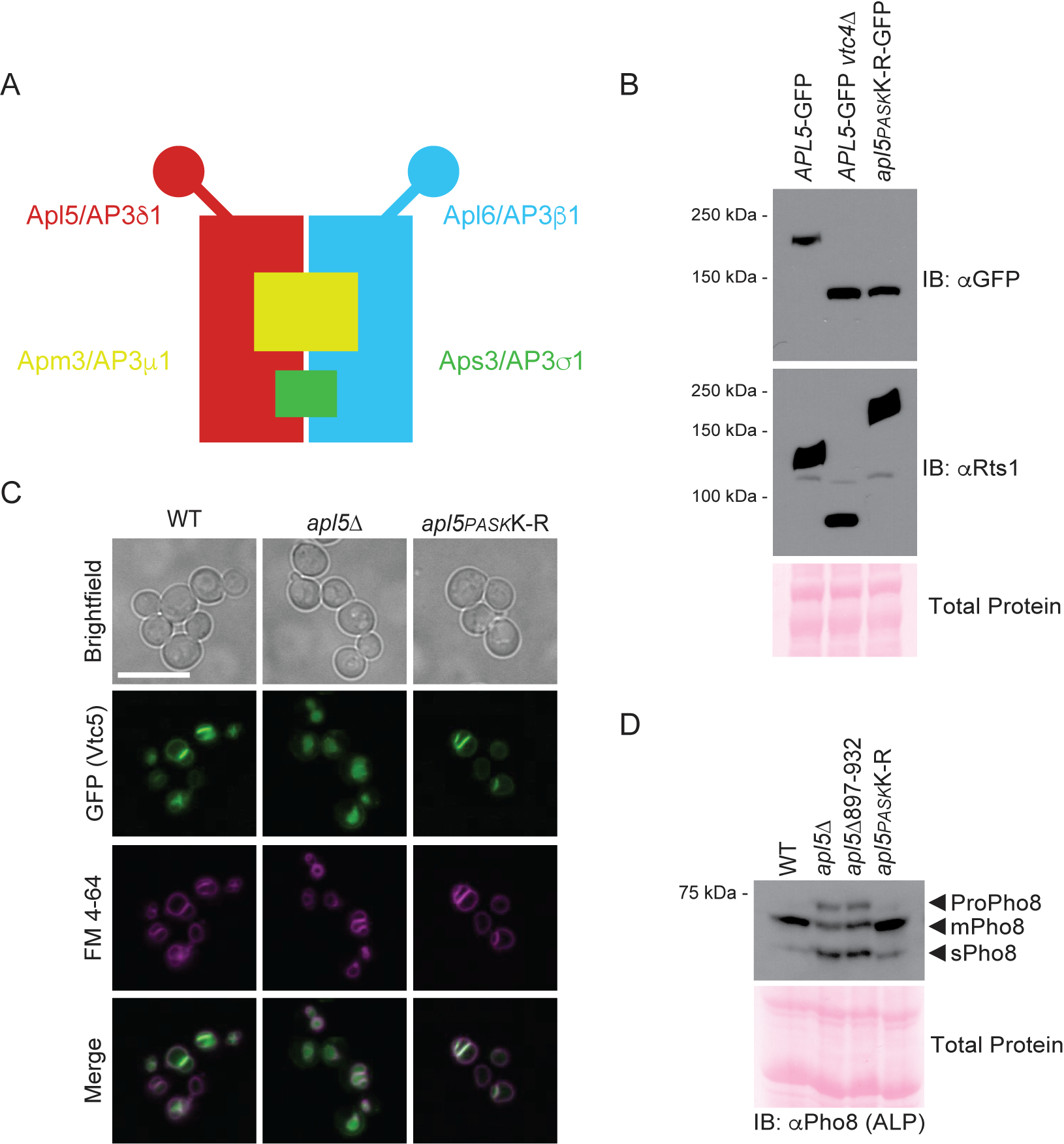

Supplement: REVISED FIG S1 [file mbio.00994-21-sf001-revised.tif]

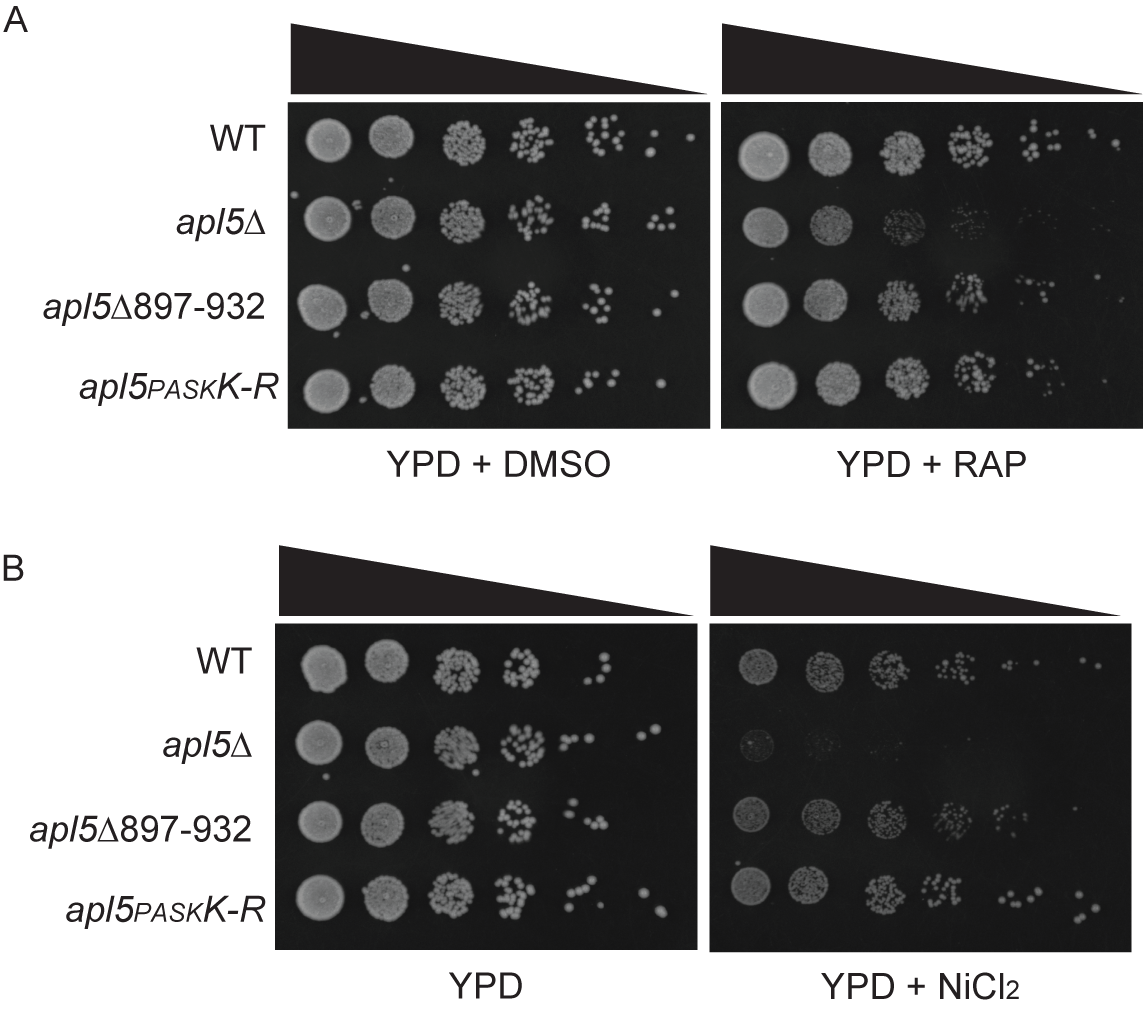

Supplement: REVISED FIG S1 [file mbio.00994-21-sf001-original.tif]

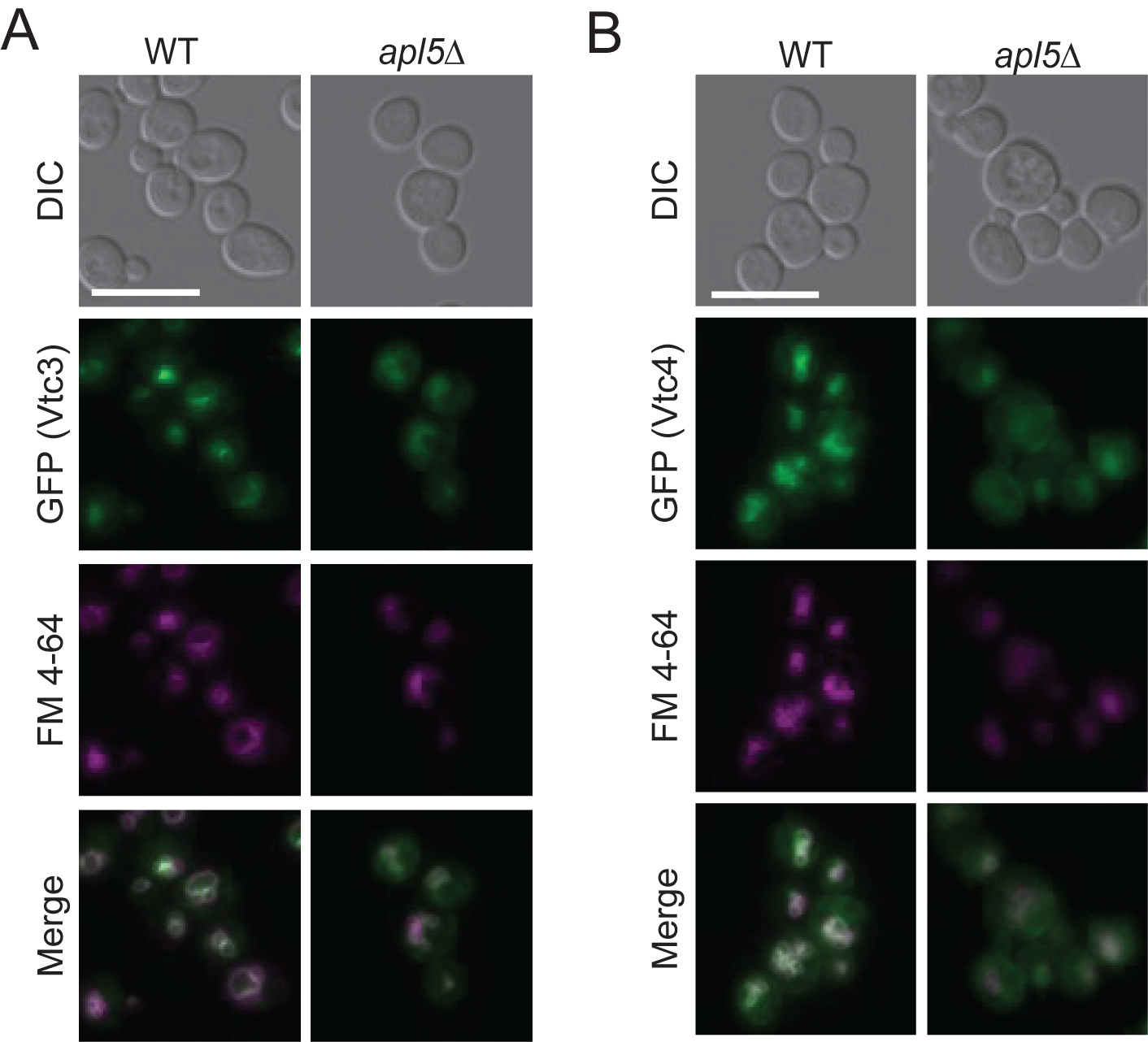

Supplement: REVISED FIG S2 [file mbio.00994-21-sf002-original.tif]

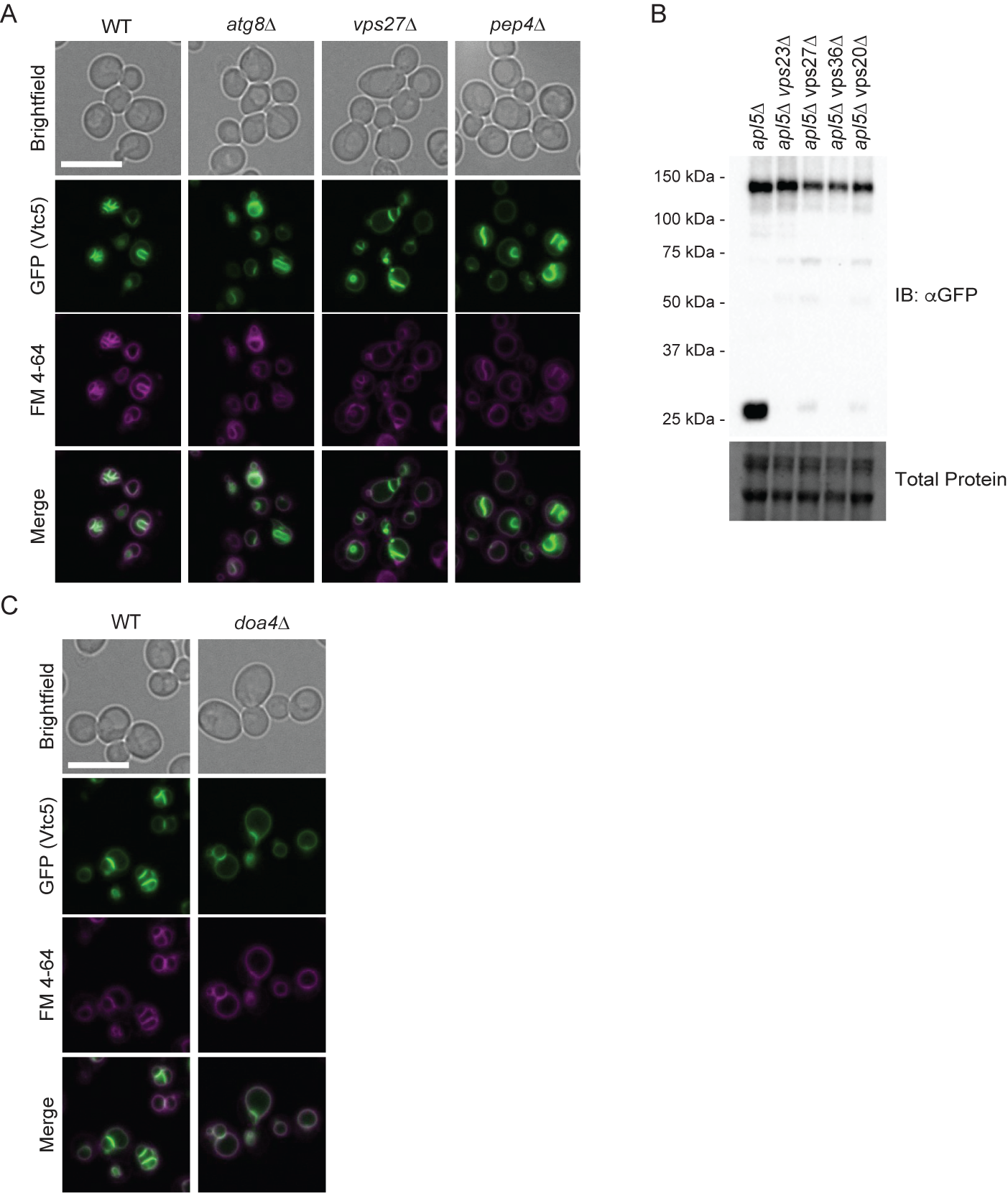

Supplement: REVISED FIG S3 [file mbio.00994-21-sf003-original.tif]
